# Society First Underestimates, Then Overestimates and Finally Ignores COVID-19: *Quo Vadis*, Cardiac Surgery?

**DOI:** 10.21470/1678-9741-2020-0460

**Published:** 2022

**Authors:** Ignazio Condello, Nicola Di Bari

**Affiliations:** 1 Department of Cardiac Surgery, Anthea Hospital, GVM Care & Research, Bari, Italy. E-mail: ignicondello@hotmail.it; 2 Division of Cardiac Surgery, Dipartimento di Emergenza e Trapianti di Organo (D.E.T.O.), University of Bari, Bari, Italy.

In this letter to the editor, we present how often culture and society can influence the course of transmission of a disease such as COVID-19, but not only. In particular, during the lockdown for COVID-19, patients suffering from heart disease with non-acute symptoms found themselves postponing the intervention as it was not urgent. In general, elective surgery is usually postponed, but necessary/emergency surgery may still be indicated. Between January 1^st^ and April 30^th^, cardiac surgery activity in public and private “COVID-free” hospitals in the Apulian region was reduced.

We treated only urgent/emergent cases or those whose treatment was considered non-postponable for more than a month. After the lockdown, from early May the cardiac surgery activity in the Apulian region was resumed, as the incidence of COVID-19 cases was zero. Cardiac surgery has undergone, like other branches of medicine, the interruption of elective procedures. The months from May to June in Puglia were characterized, as in the rest of Italy, by the reopening of public places and nightclubs, which caused an increase in contagions. However, in relation to the article “Cardiac surgery model during COVID-19 pandemic: now it's time to ramp up”, by Folesani et al. ^[1]^, we think that, in this moment, the virtuous strategies implemented by hospitals such as oropharyngeal swabs at ordinary intervals and the use of personal protective equipment for patients and healthcare personnel are likely to prevent the interruption of heart surgeries, despite the disrespect for social distancing in the reopening and underestimation of COVID-19 by some people.
